# Editorial: Hydropic Ear Disease: Imaging and Functional Evaluation

**DOI:** 10.3389/fsurg.2022.913741

**Published:** 2022-04-27

**Authors:** Shinji Naganawa

**Affiliations:** Department of Radiology, Graduate School of Medicine, Nagoya University, Nagoya, Japan

**Keywords:** magnetic resonance imaging (MRI), Meniere's disease, Endolymphatic hydrops-related disease, Gadolilnium, Hydropic ear disease

**Editorial on the Research Topic**

***Hydropic Ear Disease: Imaging and functional evaluation***

Recent developments and advances in the objective methods of evaluating endolymphatic hydrops and its related inner ear diseases have led to a better understanding of inner ear disease. Twenty-one valuable articles have been published in this issue of the research topic “Hydropic Ear Disease: Imaging and functional evaluation” on the advances in diagnostic imaging and techniques for measuring the function of the six sensory organs present in the inner ear. As of the beginning of April 2022, it has already recorded more than 47,500 views, which means that readers are very interested in this project. Fifteen out of the 21 papers report on magnetic resonance imaging (MRI) of the patients.

MRI depiction of endolymphatic hydrops in the patients with Meniere’s disease was firstly achieved in 2007 with 3D-fluid attenuated inversion recovery (FLAIR) obtained 24 h after intratympanic gadolinium contrast administration (1). A clinically feasible method using a heavily T2-weighted 3D-FLAIR after 4 hours of intravenous injection of a standard dose of gadolinium contrast agent was achieved in 2010 (2). After these developments, many technical improvements have been reported including the development of HYDROPS (HYbriD of Reversed image Of Positive endolymph signal and native image of positive perilymph Signal) technique, which utilized the subtraction of two kinds of images (3). After these developments, many institutions began to acquire the MR imaging of endolymphatic hydrops. Subsequently, an update of endolymphatic hydrops assessment using MR imaging has been proposed for the management of inner ear disease (4–7).

A number of attempts have been made to perform the assessment of endolymphatic hydrops by MRI without the use of gadolinium contrast media (8–12). However, these are unfortunately unreliable because they do not adequately distinguish between artifact and imaging findings (13–15). Recognition of the endolymphatic space on non-contrast MRI is still possible only in very exceptional cases. These exceptional cases include hemorrhage into the ampulla (16), reflux of proteinous fluid in the enlarged endolymphatic duct and sac syndrome (17), and the compositional change of perilymph due to vestibular schwannomas (18). In general, contrast-enhanced MRI evaluation of endolymphatic hydrops is the most reliable method of examination at this time.

In this research topic, Fukushima et al. have reported on the use of MRI of endolymphatic hydrops to observe the effect of positive pressure therapy in a Meniere's disease patient (https://www.frontiersin.org/articles/10.3389/fsurg.2021. 606100/full). Although this is a case report, this is a new and interesting proposal for the valuable and practical use of the MR imaging of endolymphatic hydrops.

In a review article of this research topic, that is co-authored by Topic editors, the authors tried to discuss and present a consensus on patient selection, imaging techniques, and evaluation methods at the occasion of 15 years after the invention of the MR imaging of the endolymphatic hydrops (https://www.frontiersin.org/articles/10.3389/fsurg.2022.874971/abstract).

It is expected that this issue of research topic will stimulate more interest in this field of research, encourage more researchers to enter the field, and ultimately help bring good news to patients suffering from the hydropics ear disease.
